# Using Maize Chromosome Segment Substitution Line Populations for the Identification of Loci Associated with Multiple Disease Resistance

**DOI:** 10.1534/g3.118.200866

**Published:** 2018-11-20

**Authors:** Luis O. Lopez-Zuniga, Petra Wolters, Scott Davis, Teclemariam Weldekidan, Judith M. Kolkman, Rebecca Nelson, K. S. Hooda, Elizabeth Rucker, Wade Thomason, Randall Wisser, Peter Balint-Kurti

**Affiliations:** * Dept. of Crop Science, North Carolina State University, Box 7620, Raleigh, NC 27695; † Dupont-Pioneer 7300 NW 62^nd^ Avenue P.O Box 1004 Johnston, IA, 50131-1004; ‡ Dept. of Plant and Soil Sciences, University of Delaware, Newark, DE 19716; § Department of Plant Pathology and Plant-Microbe Biology Cornell University, Ithaca, NY 14853; ** ICAR-Indian Institute of Maize Research, Indian Council of Agricultural Research, Pusa Campus, New Delhi 110 012, India; †† School of Plant and Environmental Sciences, Virginia Tech, Blacksburg, VA 24061; ‡‡ Dept. of Entomology and Plant Pathology, North Carolina State University, Box 7616 Raleigh, NC 27695; §§ Plant Science Research Unit, USDA-ARS, Raleigh NC 27695-7616

**Keywords:** Maize disease resistance, Multiple disease resistance, QTL

## Abstract

Southern Leaf Blight (SLB), Northern Leaf Blight (NLB), and Gray Leaf Spot (GLS) caused by *Cochliobolus heterostrophus*, *Setosphaeria turcica*, and *Cercospora zeae-maydis* respectively, are among the most important diseases of corn worldwide. Previously, moderately high and significantly positive genetic correlations between resistance levels to each of these diseases were identified in a panel of 253 diverse maize inbred lines. The goal of this study was to identify loci underlying disease resistance in some of the most multiple disease resistant (MDR) lines by the creation of chromosome segment substitution line (CSSL) populations in multiple disease susceptible (MDS) backgrounds. Four MDR lines (NC304, NC344, Ki3, NC262) were used as donor parents and two MDS lines (Oh7B, H100) were used as recurrent parents to produce eight BC_3_F_4:5_ CSSL populations comprising 1,611 lines in total. Each population was genotyped and assessed for each disease in replicated trials in two environments. Moderate to high heritabilities on an entry mean basis were observed (0.32 to 0.83). Several lines in each population were significantly more resistant than the MDS parental lines for each disease. Multiple quantitative trait loci (QTL) for disease resistance were detected for each disease in most of the populations. Seventeen QTL were associated with variation in resistance to more than one disease (SLB/NLB: 2; SLB/GLS: 7; NLB/GLS: 2 and 6 to all three diseases). For most populations and most disease combinations, significant correlations were observed between disease scores and also between marker effects for each disease. The number of lines that were resistant to more than one disease was significantly higher than would be expected by chance. Using the results from individual QTL analyses, a composite statistic based on Mahalanobis distance (*Md*) was used to identify joint marker associations with multiple diseases. Across all populations and diseases, 246 markers had significant *Md* values. However further analysis revealed that most of these associations were due to strong QTL effects on a single disease. Together, these findings reinforce our previous conclusions that loci associated with resistance to different diseases are clustered in the genome more often than would be expected by chance. Nevertheless true MDR loci which have significant effects on more than one disease are still much rarer than loci with single disease effects.

Genetic resistance is the most cost-effective and environment-friendly method for reducing losses in yield and quality in agricultural crops caused by plant disease. Disease resistance is often described in the literature as being either qualitative or quantitative. Qualitative disease resistance typically confers high levels of resistance and is generally controlled by a single or a few genes with major effects (Bent and Mackey 2007; Ross 1986; Van der Plank 1965). Quantitative disease resistance (QDR) typically confers partial resistance, is usually based on the effect of multiple genes and is generally believed to be much more durable in the field than qualitative resistance (Poland *et al.* 2009, Niks *et al.* 2015).

Multiple disease resistance (MDR) can refer to a host plant with resistance to more than one disease or to a gene or allele that confers resistance to more than one disease (Wiesner-Hanks and Nelson 2016). The MDR principle can be traced back at least to 1902 where a cowpea cultivar was described to be resistant to wilt and root-knot, *Neocosmospora vasinfecta* and *Heterodera radicicola*, respectively (Orto 1902). Since then, many authors have reported on successful development of lines with MDR in a great number of crops such as wheat, barley and potato (Jansky and Rouse 2003; Mitchell-Olds *et al.*, 1995; Risk *et al.*, 2013; Schnurbusch *et al.*, 2004). Multiple-disease resistant lines may contain several genes acting independently to confer resistance to multiple diseases, or single genes conferring multiple resistance to several diseases. Several examples of MDR genes are described in the literature. For example, *Mi-1* confers resistance to both aphids and nematodes in tomato (Vos *et al.*, 1998) and *Lr34/Yr18* confers resistance to leaf rust, stripe rust, steam rust, and powdery mildew in wheat (Spielmeyer *et al.* 2013). Other genes conferring MDR in wheat include *Lr46*/*Yr29*, *Lr67*, and *Yr30* (Bariana *et al.*, 2007; Rosewarne *et al.*, 2008; Spielmeyer *et al.*, 2013; William *et al.*, 2007). In this study, the genetic basis of resistance to three diseases of maize was investigated: Gray leaf spot (GLS); Northern leaf blight (NLB); and Southern leaf blight (SLB). Each of these diseases are caused by an ascomycete fungal pathogen that infects leaves of maize.

SLB is caused by the necrotrophic fungus *Cochliobolus heterostrophus* (anamorph *Bipolaris maydis*) and often occurs where maize is grown under hot and humid conditions. Irregularly shaped lesions initially from the lower to upper canopy of the plant and expand until they coalesce within leaves to cover large sections or entire leaves (White 1999). In the early 1970s, *C. heterostrophus* race T, which produces the so-called T-toxin, caused an important epidemic in the U.S., causing overall losses of approximately 12% -15% (Ullstrup 1972). After the epidemic, farmers in the U.S. reverted to growing lines that were not susceptible to *C. heterostrophus* race T. Currently, the most common race of *C. heterostrophus* in the U.S. is race O, which can cause yield losses of greater than 40% in inoculated experimental fields (Byrnes and Pataky, 1989, Fisher, Hooker, *et al.*, 1976, Gregory, Ayers, *et al.*, 1979), although losses are normally much lower due to the use of resistant hybrids. Resistance to *C. heterostrophus* race O is quantitatively inherited with primarily additive or partially dominant gene action (Holley and Goodman 1989). Numerous QTL studies have been conducted to map loci associated with SLB resistance (Balint-Kurti *et al.*, 2008b; Balint-Kurti *et al.*, 2006; Balint-Kurti *et al.*, 2007; Bian *et al.*, 2014; Carson *et al.*, 2004; Kump *et al.*, 2011; Negeri *et al.*, 2011; Zwonitzer *et al.*, 2009; Zwonitzer *et al.*, 2010).

NLB caused by the hemibiotrophic fungus *Setosphaeria turcica* (anamorph *Exserohilum turcicum*) is found throughout the world in humid areas with moderate temperatures. Like SLB, the disease initially develops on the lower canopy and spreads to leaves of the upper canopy, producing expansive cigar-shaped lesions. In some U.S. regions, yield loses can be greater than 50% (Raymundo and Hooker 1981; Tefferi *et al.*, 1996). Both qualitative and quantitative forms of phenotypic variation in resistance to NLB have been characterized. The qualitative resistance genes *Htl*, *Ht2*, *Ht3* confer resistance to specific races of NLB (Bentolila *et al.*, 1991; Hooker 1963; Simcox and Bennetzen 1993; Yin *et al.*, 2003). One of these race-specific genes, *Htnl*, was recently identified as a wall-associated receptor-like kinase (Hurni *et al.*, 2015). Because qualitative resistance is generally less durable in the field, some research has focused on quantitative disease resistance, revealing a polygenic architecture composed primarily of additive effects (Balint-Kurti *et al.*, 2010; Poland *et al.*, 2011; Chung *et al.*, 2010, 2011, Jamann *et al.* 2016).

GLS is caused by the related species *Cercospora zeae-maydis* and *C. zeina* (Meisel *et al.* 2009; Ward *et al.* 1999) which both have a necrotrophic lifestyle. Most reports of GLS describe its occurrence in the United States (Ward *et al.*, 1997, Tehon and Daniels 1925) and Africa (Dunkle and Levy 2000), with some reports of GLS in China and Latin America (Ward *et al.*, 1999). Disease development is favored in temperate, humid conditions. Similar to SLB and NLB, symptoms are first observed on the lower leaves as a mixture of small, irregularly shaped spots and semi-rectangular lesions which spread up the plant while lesions expand parallel to the veins of the leaf (Ward *et al.*, 1999). Losses due to GLS can range from 10 to 25% annually, but in severe cases this value can reach 50% (Freppon *et al.*, 1996; Ward *et al.*, 1999). Residues from continuous cropping managed under conservation tillage also promote the development of the disease (Ward *et al.*, 1999). Phenotypic variation in resistance to GLS in maize is generally quantitative, with a polygenic architecture primarily composed of additive allele effects (Lyimo *et al.*, 2011; Zhang *et al.*, 2012 Balint-Kurti *et al.*, 2008a; Derera *et al.*, 2008; Gordon *et al.*, 2006; Juliatti *et al.*, 2009; Lennon 2014; Zwonitzer *et al.*, 2010, Benson *et al.* 2015).

While the infection processes of the pathogens causing SLB, NLB, and GLS have several distinguishing features, they share some aspects (Beckman and Payne 1982; Jennings and Ullstrup 1957). For instance, they all penetrate the leaf and, in early stages, grow in living tissue, but ultimately derive their nutrition from dead tissue. Gene(s) affecting some of these shared pathogenesis processes might be expected to confer MDR. Indeed, using multivariate statistical analysis of data on SLB, NLB, and GLS resistance scored in a panel of maize inbred lines developed for high-resolution association mapping (Flint-Garcia *et al.* 2005), Wisser *et al.* (2011) found moderately high positive genetic correlations between resistances in all pairwise combinations. Because linkage disequilibrium (LD) in that panel is low (Flint-Garcia *et al.* 2005), the authors inferred that a component of the variation in MDR was attributable to alleles with pleiotropic effects. Single nucleotide polymorphisms (SNPs) within a glutathione *S*-transferase gene on chromosome 7 were associated with MDR.

The goal of the current study was to produce chromosome segment substitution lines (CSSL) in which a whole genome tiling path of introgressions from MDR lines is captured in MDS genomic backgrounds and to use these populations to determine if loci underlying MDR could be identified. The rationale for choosing this approach was twofold; First we reasoned that the effects of some resistance loci might be best observed in a susceptible background and second, we wanted to identify MDR NIL lines that could be used directly in subsequent studies probing the mechanisms of resistance. Four top-ranking MDR lines (NC304, NC344, Ki3 and NC262) identified in Wisser *et al.* (2011) were used as donor parents and two low-ranking MDS lines (Oh7B, H100) were used as recurrent parents to produce eight CSSL populations.

## Materials and Methods

### Plant materials, populations

Six inbred maize lines were used to produce eight different populations. The parental materials were: inbred line H100 (developed by Indiana Agricultural Experiment Station from N28 x H91; Dudley *et al.*, 1991), inbred line Ki3 (developed from Suwan-1(S) C4 (Thailand); Liu *et al.*, 2003), inbred line NC262 (developed by North Carolina State University from McNair 14 x McNair 18; Nelson *et al.*, 2016), inbred line NC304 (developed by North Carolina State University from (Pioneer X105A x H5) x H101; Nelson *et al.*, 2016), inbred line NC344 (developed by North Carolina State University from (McNair 14x18)^2 x [(NC246 x NC248) x C103]; Nelson *et al.*, 2016), and inbred line Oh7B (developed in Ohio from (Oh07 × 38-11) x Oh07; Liu *et al.*, 2003).

**Figure 1:**
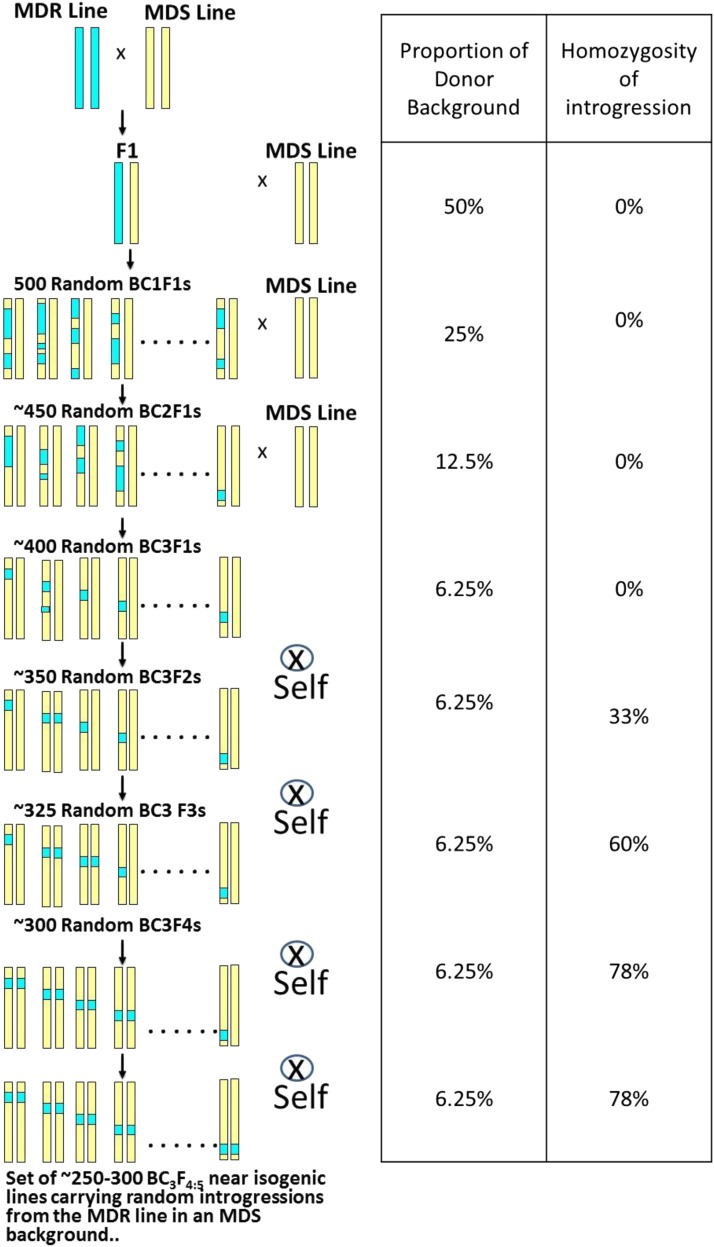
Scheme used to produce all chromosome segment substitution lines populations in this study.

Eight chromosome segment substitution populations were created by crossing four MDR donor lines, Ki3, NC262, NC304, and NC344 as females, with two multiple disease susceptible recurrent lines, H100 and Oh7B as male. After the F1 was obtained, three generations of backcrosses with H100 or Oh7B as males were performed, followed by four generations of self-pollination via single-seed descent to obtain BC3F4:5 lines (Figure 1). The F4:5 lines were subsequently increased by sib-mating within each line. An identification code was ascribed to each line, starting with the prefix DRIL (for “Disease Resistance Introgression Line”) followed by a population code for each cross based on line codes: (H100 = 2, Ki3 = 3, NC262 = 5, NC304 = 6, NC344 = 7, Oh7B = 8); where 32, 52, 62, and 72 correspond to Ki3/H100, NC262/H100, NC304/H100, and NC344/H100, respectively, and 38, 58, 68, and 78 correspond to Ki3/OH7B, NC262/OH7B, NC304/OH7B, and NC344/OH7B, respectively. In each case the code number for the donor (female) parent is listed first and the number for the recurrent (male) parent is listed second, followed by a numerical identifier for each line (*e.g.*, DRIL32-001, DRIL32-002, etc.).

### Generation of genotypic data

Young leaf tips from six plants per line were collected and lyophilized for DNA extraction and genotyping. DNA extraction was performed using the commercially available Gentra Puregene Tissue Kit. The final DRIL populations were genotyped with the Pioneer Illumina publicplex platform using 765 single nucleotide polymorphism (SNP) markers (Jones *et al.*, 2009). The genetic map locations used were based on the IBM4 map (Fu *et al.*, 2006). Files S1-S8 have details of all the markers used in each population.

On average 3.1% of the marker data were missing. Missing data imputation was performed using a set of simple algorithms in Microsoft Excel based on flanking markers. For each line, if a data point was missing for a marker (X), and if the parental genotypes for the two flanking markers were identical, then the missing data were imputed to be the same parental genotype as the flanking markers. This imputation criteria was also used for up to two consecutive missing marker values (*e.g.*, consecutive marker calls AA, XX, XX, AA becomes AA AA AA AA, where X is missing data and A is a parental allele). If the flanking markers differed, then the missing markers were imputed as the recurrent parental allele.

The BC_3_F_4:5_ lines are expected to carry 6.25% introgressed DNA derived from the donor genome, and 78% of that introgressed DNA is expected to be present as homozygous introgressions (Figure 1). The genotypic data were systematically evaluated to identify and eliminate lines that substantially deviated from these expectations. In these cases, it was likely that the lines had been the product of one or more contaminant pollinations.

To eliminate lines that were not likely the product of the given crossing scheme, DRILs were eliminated if they exceeded two standard deviations from the median value of either: (i) the total percentage of markers in a line that were heterozygous and the number of introgression segments in a line; (ii) the percentage of donor parent alleles detected for each line; or (iii) any combination of (i) and (ii).

A similar process was performed to identify markers with anomalous scores. For each DRIL population, any given marker locus would be expected to carry donor parent alleles at approximately 6.25% of loci assessed and 78% of these donor alleles would be expected to be present in the homozygous state. Additionally, marker loci would be expected to have similar genotypes at genetically linked marker loci most of the time. After anomalous lines had been eliminated, two parameters were calculated from the remaining lines in each population: the percentage of lines that were heterozygous for a particular marker and the percentage in each population that a marker was different from its flanking marker (we refer to this metric as percent unlinked marker). If a marker presented a value in any of these two parameters that was higher than the median plus two standard deviations, it was eliminated. The exceptions to this were when the marker was the final marker on a chromosome and when a high percentage of unlinked markers was caused by the anomalous behavior of the neighboring marker. To determine this, the most anomalous markers (top third) were eliminated first and then the analysis was performed again to reevaluate the remaining markers each time.

### Inoculum Preparation and Inoculation Procedure

Sorghum grains were soaked from 3 to 4 days in water, placed in 1 L flasks, and autoclaved for one hour (120 PSI and 121°). Autoclaved grain was inoculated with either *C. heterostrophus*, *S. turcica*, *or C. zeae-maydis*. The fungus grew at room temperature (23-25°) for approximately 10, 14 or 21 days for *C. heterostrophus*, *S. turcica*, *or C. zeae-maydis* respectively until the sorghum was colonized with the fungus. The sorghum was air-dried and stored at 4°. Each isolate of each fungus was grown and dried separately. The dried sorghum infested with isolates of the same species was thoroughly mixed just prior to inoculation. The same mixture was used to inoculate the entire trial. Twenty-five to thirty-day old maize plants were inoculated by adding 6 to 10 infested sorghum kernels placed into the whorl of each maize plant.

### Disease evaluations

All trials used an augmented lattice design. The number of entries varied between each population and are shown in Table 1. Two environments were used for every assessment and two replications were used in every environment except for the case of NC262/H100 where only one replication was planted in one of the years in the GLS evaluation. The number of blocks within each replication varied depending on the number of lines in the population but was at least 10 in every case. A recurrent parent check was included in each block and the resistant donor parent was also planted several times in each replication.

**Table 1:** Number of lines and markers and associated parameters for each population

Population	Ki3/H100	Ki3/Oh7B	NC262/H100	NC262/Oh7B
Lines	265	204	195	111
Markers	245	239	271	209
^1^Percent Recurrent Parent	93.5	92.7	91.3	85.2
^2^Percent Donor Parent	6.5	7.3	8.7	14.8
^3^Percent Heterozygous	0.8	1.2	1.6	3.1
^4^Percent Segregating Introgressions	12.2	16.7	19.2	19.8
^5^Number of Introgressions	6.3	6.9	7.8	10.5
^6^Percent Donor Genome	100	98	100	100

Population	NC304/H100	NC304/Oh7B	NC344/H100	NC304/Oh7B
Lines	252	108	258	218
Markers	270	254	270	241
Percent Homozygous Recurrent Parent	93.1	91	93.2	92.05
Percent Homozygous Donor Parent	6.9	9	6.8	7.5
Percent Heterozygous	1.6	1.2	1.3	1
Percent Segregating Introgressions	19.9	12.4	19.6	13.7
Number of Introgressions	6.7	7.6	6.4	5.9
Percent Donor Genome Represented	100	100	100	100

1 The total number of markers scored as homozygous for the recurrent parent allele plus half of the total number of markers scored as heterozygous, divided by the total number of markers used for the population, multiplied by 100.

2 The total number of markers scored as homozygous for the donor parent allele plus half of the total number of markers scored as heterozygous, divided by the total number of markers used for the population multiplied by 100.

3 Number of markers scored as heterozygous divided by the total number of markers multiplied by 100.

4 Number of markers scored as heterozygous divided by the total number of markers scored as homozygous or heterozygous for the donor parent allele multiplied by 100.

5 Number of segments that contain homozygous or heterozygous donor parental allele.

6 Number of markers scored as homozygous or heterozygous for the donor parental allele contained within the population, divided by the total number of markers multiplied by 100. The population mean of these statistics are shown. For % of introgression the range is also shown.

Field evaluations for SLB disease were performed during the summers of 2012 and 2013 for the NC262/H100 and NC262/Oh7B populations, and in 2014 and 2015 for the Ki3/H100, NC304/H100, NC344/H100, Ki3/Oh7B, NC304/Oh7B, and NC344/Oh7B populations. All SLB phenotypic evaluations were performed at Central Crops Research Station (CCRS) in Clayton, NC. Experiments were planted in 1.8 m single rows with a 0.9 m row width using 10 seeds per plot. To assure high disease pressure, SLB inoculations were carried out 30 days after planting using a mixture of three isolates of *C. heterostrophus* including 2-16Bm, Hm540 (Carson 1998), and an unnamed isolate provided by Syngenta. Visual scores of the disease were taken three or four times at intervals of eight to ten days starting two weeks after anthesis (when 50% of the plants in a row were shedding pollen) on a scale 1 to 9 where 1 = 100% leaf area affected by the pathogen and 9 = no disease. For each plot, days from planting to anthesis, plant height, and ear height were recorded.

Field evaluations for NLB disease were performed during summer of 2013 for NC262/H100 and NC262/OH7B population at CCRS (environment 1) and at Aurora, NY, Cornell University NY (environment 2). Ki3/Oh7B, NC304/Oh7B, and NC344/Oh7B populations were evaluated for NLB at CCRS in 2014 and 2015. Ki3/H100, NC304/H100, and NC344/H100 were evaluated in 2014 at CCRS. At CCRS trials were planted as described above. In Aurora NY, trials were planted in 2.4 m single rows with a 1 m row width using 10 seeds per plot. To assure high disease pressure, NLB inoculations were performed using several *S. turica* isolates (ET238A, ET471A-1, ET30A, ET3A, ET28A, ET257A, Cairo05, 235A, race 0 from Syngenta, Race1 from Syngenta, ET252A, ET28A, ET30A, ET222A) 30 to 45 days after planting in Clayton. In New York, inoculations were performed using a local isolate NY001 (race 1). At Clayton, three to four visual scores of the disease were taken at intervals of eight to ten days starting two weeks after anthesis. At NY, scores were taken two times, at an interval of one week, starting two weeks after anthesis. For each plot, days to anthesis was also recorded. At each location, the percentage of diseased leaf area was used to score the disease. For the purpose of correlation analysis, NLB disease scores (from 0 to 100%, where 0 is the most resistant) were converted to a scale similar to the SLB and GLS phenotyping system. Field evaluations for GLS disease were performed during the summers of 2012 and 2013 at Andrews, NC for the NC262/H100 and NC262/OH7B populations, and at Andrews, NC and at the Virginia Tech Kentland Farm, near Blacksburg, VA, during the summer of 2014 for the Ki3/H100, NC304/H100 and NC344/H100 populations. The Ki3/OH7B, NC304/OH7B, and NC344/OH7B populations underwent GLS phenotypic evaluations during summers of 2014 and 2015 at Andrews NC and Blacksburg VA respectively. Trials were planted in 4 m single rows with a 1 m row width using 16 seeds per plot. The fields used in Andrews contained infected plant debris from the previous season and therefore had a very high inoculum disease pressure and artificial inoculation was not required. In Virginia, inoculations were performed using a mixture of 10 field-derived *C. zeae-maydis* isolates 30 to 45 days after planting. Visual scores of the disease were taken twice at an interval of 10 days starting two weeks after anthesis. Ratings ranged from 1 to 9 where 1 = 100% leaf area affected by the pathogen and 9 = no disease. Days toanthesis was also recorded in Andrews for the NC262/H100 and NC262/OH7B populations.

The symptoms of SLB, NLB and GLS are distinct from each other. In every case in every experiment we observed almost exclusively the symptoms expected of the targeted disease until late in the season (after completion of data collection when the plants had started to senesce).

### Statistical Analysis

Statistical analysis of phenotypic data were performed using R (R core development team 2008). Q-Q, residual, and distribution plots were also produced to determine normality and variance homogeneity. The standardized area under disease progress curve (sAUDPC, a quantitative summary of disease severity over time; Simko and Piepho 2012) was calculated for each genotype and each disease based on a minimum of two and maximum of four sequential ratings. Using lme4 (Bates *et al.*, 2015), a univariate linear mixed model was fit to the data for each disease:

$$sAUDPC=B\left(R\right)+B\left(R^{*}E\right)+G+E+G^{*}E+\varepsilon ;$$

where, sAUDPC is the response variable as described above, genotype (G) is a fixed effect, block [B(R)], replication $\left[B\left(R^{*}E\right)\right]$, environment (E) and genotype-by-environment ($G^{*}E$) is a random effect and ε is a random residual effect. Least square means (LSMeans) for each genotype within each disease were obtained and the lines that had LSMeans significantly different from the recurrent check H100 or Oh7B were identified using a multiple comparison test, mulcomp/glht in R, in which a multiple test correction due to heterogeneous or unequal variances among groups was accounted.

Heritability was estimated on an entry mean basis (Holland *et al.* 2003). This was determined for each population-disease combination. Variance components used to compute hertitability were estimated from a model similar to the one described above, except that genotype was fit as a random effect in order to also estimate the genotypic variance.

Pairwise Pearson correlations between LSMeans for each disease within each population were calculated. A Chi square test was used to show test whether the number of multiple disease resistant lines identified in each genetic background (H100 and Oh7B) was higher than would be expected due to random chance. Fisher’s exact test was used to test the same thing for individual populations as Chi square does not deal properly with smaller numbers (<5) (Bearden *et al.* 1982).

### QTL analyses

For each DRIL population and disease, QTL analysis was performed using the QTL IciMapping software (Meng, Li, *et al.*, 2015), employing the option for non-idealized CSSL QTL analysis (CSSL with more than one introgression). For this analysis, all recurrent homozygous genotypes, heterozygous genotypes and missing genotypes were coded as 0 and homozygous donor genotypes were coded as 2. The program allows for marker multicollinearity control, and for this a “by condition number” of 1000. A RSTEP-LRT method, which is a likelihood ratio test based on stepwise regression, was used for QTL mapping. Stepwise regression was used to select the most significant markers and a likelihood ratio test was used to calculate the LOD scores for each marker. The probability threshold used for stepwise regression was 0.01 while LOD thresholds were determined by permutation testing (1000 iterations) with a type I error of 0.05.

Outputs of QTL analysis included LOD values, percentage of variance explained, and additive effects of the marker. Additive effects of NLB were converted to the same SLB/GLS scale. Pairwise correlations between the marker additive effects were calculated. The IBM4 genetic map was used as a guide for placing the markers on the maize genetic map (Fu *et al.*, 2006; Jones *et al.*, 2009).

### Composite Statistic

Following Rousseeuw and Zomeren (1990), Rousseeuw (1985) and Lotterhous *et al.* (2017), a composite statistic based on Mahalanobis distance (*M_d_*) was used to identify marker associations that represent multivariate outliers in three-dimensional space (*i.e.*, three traits). For each trait, LOD scores were converted into p-values: $P\left(\mathrm{LOD}\right)=.5\times \left(X_{1}^{2}>2\ln10\times \mathrm{LOD}\right)$ (Nyholt 2000). Using the negative log_10_ of the p-values for the three traits, minimum covariance determinant was used to compute the trait-wise covariance matrix for robust detection of outliers (Lotterhos *et al.* 2017). The analysis was performed using the OutlierMahdist function of the rrcovHD package v. 1.4-4 (Todorov and Filzmoser 2009).

*M_d_*^2^ values follow a $X^{2}$ distribution with degrees of freedom equal to the number of dimensions (Rousseeuw and Zomeren 1990), so the probability of *M_d_*^2^ for each marker was computed. The “BH” method (Benjamini and Hochberg 1995) of the p.adjust function in R was used to adjust for multiple marker tests per population. Significant multivariate outliers were declared at a 1% false discovery rate. In addition, because a number of significant outliers were identified despite having sub-threshold LOD scores for all three traits, an additional higher threshold for significance was set based on whether the *M_d_*^2^ value was significant and the marker LOD score from QTL mapping was greater than the LOD threshold for at least one of the three traits. Note: the LOD threshold varied, as it was computed by permutation for each population-disease combination (see above). Custom R scripts were used to make figures for the results from analyses using the composite statistic.

### Statement of data and reagent availability

All lines are available on request form the corresponding authors. The populations are currently being deposited at the Maize Genetics Stock Center (http://maizecoop.cropsci.uiuc.edu/). All genotypic and phenotypic information used in this work is included in the supplementary files. Supplemental material available at Figshare: https://doi.org/10.25387/g3.7265756.

## Results and Discussion

Eight different CSSL DRIL populations were created (Figure 1) in which four multiple disease resistant (MDR) donor parent maize lines (Ki3, NC262, NC304, and NC344) were crossed as females with two multiple disease susceptible (MDS) recurrent parent lines (H100 and Oh7B) as males.

Each population was assessed in disease-specific nurseries for resistance to SLB, NLB, and GLS in two environments with two replications per environment. The phenotypic distributions were, as expected, centered around the recurrent parent phenotype in each case, with the distributions skewed slightly toward resistance (Figure S1). Each DRIL was genotyped using 765 SNP markers. After quality control to eliminate anomalous DRILs, 1611 DRILs from all populations were maintained for further analysis (Table 1). Following quality control of the marker data, 209 to 271 polymorphic markers were retained in each population (Table 1). Based on imputed genotypes, the entire donor parent genome was captured by introgressions within each population except for the Ki3/Oh7B population in which 98% of the Ki3 genome was represented (Table 1).

Exploratory visual data analysis of Q-Q plots, residual plots and spatial field plots of phenotypic values did not reveal any obvious deviations from normality in the data distribution or obvious spatial field effects (not shown); a linear mixed model with normally distributed random variables and without spatial effects was fit to the data. Plotting LSMean estimates for each population-disease-environment combination indicated that donor parental lines were, as expected, highly resistant for all diseases and that the recurrent parental lines were always close to the mean of the entire population. In three out of 48 population-disease-environment combinations assessed, namely the NC304/H100, NC344/H100, and Ki3/H100 populations evaluated for NLB in Aurora NY, the data were deemed unusable based on inconsistent phenotypes for the repeated parental checks. In these cases, we relied on data from just one environment with two replications.

Significant differences were always observed between the donor and recurrent parents for each disease and for each population (Table 2). In every population, some DRILs were significantly more resistant than the recurrent parent for each disease (File S9). However, no significant transgressive segregation was identified. In other words, we did not identify any DRILs that were significantly more resistant than the resistant parent or significantly more susceptible than the susceptible parent (Figure S1, File S9).

**Table 2:** LSMeans for donor and recurrent parents for each population

^1^Population	Ki3/H100	Ki3/Oh7B	NC262/H100	NC262/Oh7B
**SLB Don**	8.0***	8.0***	7.8***	6.9***
**SLB Rec**	4.6	7.8	4.4	5.7
**NLB Don**	12.1***	12.8***	9.9***	15.5***
**NLB Rec**	53.4	52.4	68.4	58.9
**GLS Don**	8.1***	8.2***	7.7***	6.9***
**GLS Rec**	6	5.9	4.3	4.6

Population	NC304/H100	NC304/Oh7B	NC344/H100	NC344/Oh7B
**SLB Don**	8.0***	7.9***	8.0***	7.9***
**SLB Rec**	4.8	5.8	4.6	5.8
**NLB Don**	10.4***	12.7***	6.6***	9.9***
**NLB Rec**	61.8	50.5	47.7	55.6
**GLS Don**	8.1***	8.3***	8.0***	8.0***
**GLS Rec**	5.9	5.9	6	5.7

1 Don = donor, Rec = recurrent. Significant differences between donor and recurrent parent are represented by *** at a level of 0.001. In each population, first line is the donor and the second is the recurrent parent. SLB and GLS scored on a 1-9 scale with 9 being resistant. NLB scored on a 0–100% scale with 0 being resistant.

Pearson correlation coefficients between replications within environments varied from 0.17 to 0.73 for SLB, 0.23 to 0.63 for NLB, and 0.25 to 0.62 for GLS (Table S1). Similarly, correlations between environments were moderate but significant and varied between 0.35-0.74, 0.39-0.65, and 0.33-0.66 for all SLB, NLB, and GLS experiments respectively (Table S1). It should be noted that CSSL populations display relatively low levels of line-to-line genetically-determined phenotypic variation by design, since the DRILs were on average ≈94% similar to each other. This lack of phenotypic variation leads to lower levels of correlation and heritability than would be expected in other similarly-sized types of bi-parental mapping populations (*e.g.*, recombinant inbred lines). Nevertheless, heritability on an entry mean basis was relatively high for most of the traits: among all populations, heritability for SLB ranged between 0.65 and 0.85; heritability for NLB ranged between 0.67 and 0.83; and heritability for GLS ranged between 0.59 and 0.82 (Table 3).

**Table 3:** Heritabilities on an entry mean basis for each population and disease

Population/ Disease	Ki3/	Ki3/	NC262/	NC262/	NC304/	NC304/	NC344/	NC344/
	H100	Oh7B	H100	Oh7B	H100	Oh7B	H100	Oh7B
**SLB**	0.85	0.80	0.65	0.82	0.85	0.81	0.83	0.76
**NLB**	0.67	0.76	0.74	0.71	0.83	0.77	0.75	0.75
**GLS**	0.63	0.61	0.76	0.82	0.73	0.65	0.64	0.59

Pairwise correlation coefficients between LSMeans for different diseases measured in each population were significant in most of the cases. Among the 24 comparisons made (three pairwise comparisons in each of eight populations), 21 were significant at a *P* < 0.05 (Table 4). Significant correlation coefficients between SLB and NLB ranged from 0.15 to 0.35 among eight populations. Significant correlations coefficients between SLB and GLS ranged from 0.17 to 0.53 among six populations. Significant correlation coefficients between NLB and GLS ranged from 0.18 to 0.47 among seven populations. These results support the hypothesis that the same genes or linked genes contribute to MDR.

**Table 4:** Pairwise correlations coefficients and *p*-values for combination of diseases LSMeans scores

Population	^1^Ki3/H100 (N = 265)		Ki3/Oh7B (N = 207)		NC262/H100 (N = 194)		NC262/Oh7B (N = 111)	
**Disease**	**NLB**	**GLS**	**NLB**	**GLS**	**NLB**	**GLS**	**NLB**	**GLS**
**SLB**	0.24***	0.53***	0.35***	0.37***	0.16*	0.40***	0.21*	0.17*
**NLB**	.	0.23***	.	0.47***	.	0.09	.	0.27**

Population	NC304/H100 (N = 251)		NC304/Oh7B (N = 108)		NC344/H100 (N = 258)		NC344/Oh7B (N = 216)	
**Disease**	**NLB**	**GLS**	**NLB**	**GLS**	**NLB**	**GLS**	**NLB**	**GLS**
**SLB**	0.35***	0.53***	0.24*	0.35***	0.15*	0.41***	0.21**	0.11
**NLB**	.	0.18**	.	0.30**	.	0.18**	.	0.18**

1 Significant p-values are represented by ***, **, *, and * at a level of 0.001, 0.01, 0.05, and 0.1 respectively.

Using a 253-line diverse association mapping population, Wisser *et al.* (2011) reported significant pairwise correlation coefficients between these same three diseases that ranged from 0.55 to 0.67. Those correlations are somewhat higher than those obtained in the present study, probably due to the fact that the panel was much more phenotypically diverse than the populations used in this study. Lennon (2014) used several CSSL populations derived from the commonly used maize lines B73 and several teosinte accessions that were constructed in a similar way to the populations used in the present study. Similar to our findings using CSSL populations, correlations between LSMeans for resistance to SLB and GLS ranged between 0.23 and 0.51, with all of them being significant at level of 0.10.

Fisher’s exact test was performed to determine whether the number of MDR lines (*i.e.*, lines that were significantly more resistant than the recurrent parent for more than one disease) in each population was higher than would be expected by chance. In other words, we tested the hypothesis that resistance to one disease is genetically independent of resistance to another disease. In summary, among 24 tests for combinations of two diseases, six were significant at *P* < 0.01, seven at *P* < 0.1, and 11 were not significant (Table 5). Similarly, among eight tests for MDR to three diseases, six were significant with *P* < 0.0001, and two were not significant. Chi-square tests were conducted on the frequencies of MDR lines within all the four DRIL populations derived from H100 and also from the four derived from Oh7B inbred lines. For the H100 set, all tests were significant. For the Oh7B set, three out of four tests were significant (Table 5). These results suggest that, in most cases, a line that is resistant to one disease has an increased likelihood of being resistant to another disease. Such multiple disease resistance could be caused by shared genomic regions contributing to resistance via coupling phase linkage of donor alleles conferring resistance to different diseases or via the pleiotropic effects of the same sequence variant.

**Table 5:** Results for Fisher exact test and Chi square test to determine whether multiply disease resistant lines are present at higher levels than would be predicted given the frequencies of lines resistant to each single disease

Population	Ki3/	Ki3/	NC262/	NC262/	NC304/	NC304/	NC344/	NC344/	H100_	Oh7B_
	H100	Oh7B	H100	Oh7B	H100	Oh7B	H100	Oh7B	Pooled	Pooled
^1^**Pop Size**	265	207	194	111	251	108	258	216	968	642
^2^**SLB # Sig_lines**	74	40	6	37	101	38	49	23	230	138
**NLB # Sig_lines**	17	27	2	2	9	16	12	6	40	51
**GLS # Sig_lines**	17	4	42	38	32	1	12	10	103	53
^3^**SLB/NLB**	^4^9*	16***	1*	2	7*	9*	3	0	20***	27***
**SLB/GLS**	13***	4**	6***	14	28***	1	6*	1	53***	20**
**NLB/GLS**	2	3**	2*	2	4*	0	1	1	9*	6
**SLB/NLB/GLS**	2***	3***	1***	2***	4***	0	1***	0	8***	5***

1 Number of lines in the population.

2 Number of lines that were statistically different than the recurrent parent for the indicated disease.

3 Number of lines that were statistically different than the recurrent parent for the indicated combination of diseases.

4 Significant p-values are represented by ***, **, *, and * at a level of 0.001, 0.01, 0.05, and 0.1 respectively. For combinations of diseases, significance implies that there were more lines significantly resistant to multiple diseases than was expected based on the proportions that were significantly resistant to each single disease.

**Figure 2:**
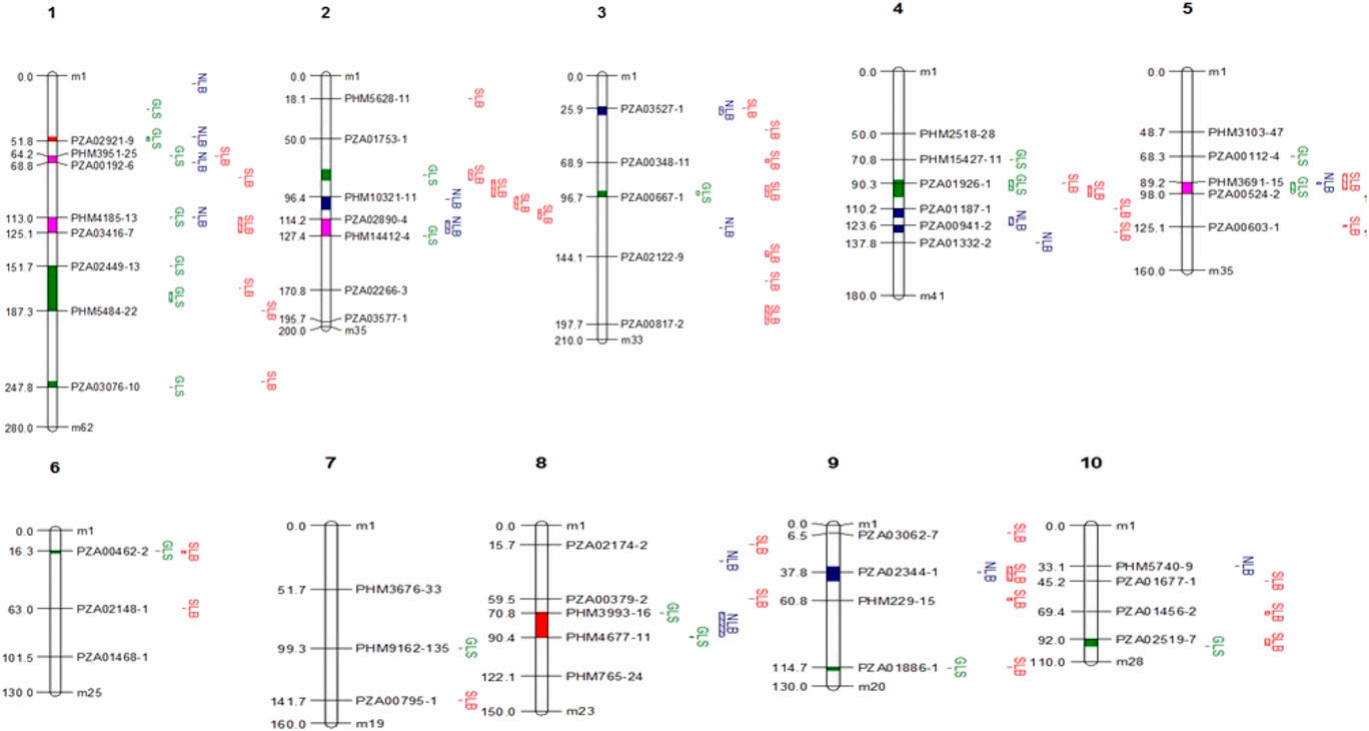
QTL detected for single, pairwise or threewise disease resistance (chromosomes 1,2, and 3). Colored segments on chromosomes represent regions associated with more than one disease though not necessarily in the same population (red = NLB/GLS, blue = SLB/NLB, green = SLB/GLS, and fuchsia = threewise). Detailed parameters for these QTL are available in Table S4.

The DRIL populations were used to map loci associated with each disease, and a composite statistic was used to identify potential MDR loci. Based on single trait analysis, a total of 56, 20, and 28 QTL of small to moderate effect were detected for SLB, NLB, and GLS respectively over all the populations (Table S2, Figure 2). The number of QTL observed among populations varied between 2 and 18. The maximum number of QTL were detected in the NC344/H100 population (18 QTL: 7, 4, and 7 for SLB, NLB, and GLS respectively), followed by the NC304/H100 population (17: 11, 0, and 6); the lowest numbers of QTL were detected in the NC262/Oh7B (2: 0, 2, and 0) population, likely due in part to the relatively small number of lines in this population.

For SLB, QTL were detected on all chromosomes, with additive effects ranging between -0.32 and 0.46 (on our 1-9 scale, with a positive value indicating that donor allele increases resistance and vice-versa). For NLB, QTL were detected on all chromosomes except 6 and 7, and additive effects were small to moderate and varied between -6.15 and 3.92 (on a scale from 0–100% where 0 is the most resistant with a negative value indicating that donor allele increases resistance). For GLS, QTL were detected on all chromosomes, with additive effects ranging between -0.25 and 0.35 (on a scale from 1-9, the same as SLB).

Pearson correlations between marker additive effects for different diseases were calculated. From 24 correlations (from 8 populations and 3 diseases), 13 were significant at a *P* < 0.05 (Table 6). These results are again congruent with the hypothesis that there exists a shared component of the genetic basis of resistance to these three diseases. Indeed, four markers exceeded the corresponding LOD threshold for two diseases in four different populations (Ki3/H100 [NLB-GLS]; Ki3/Oh7B [NLB-GLS]; NC304/H100 [SLB-GLS]; NC344/H100 [SLB-GLS]), but none exceeded the threshold for all three. In some cases, however, the LOD score of a marker was above the significance threshold for one disease, but just below the threshold for another disease. An example of this can be observed in the Ki3/H100 population on chromosome 9 at 114.68 cM where the marker had a LOD score for SLB of 5.6, well above the threshold (3.2), and not far below for NLB (threshold = 3.7, markers LOD = 2.9) and GLS (threshold = 3.03, markers LOD = 2.3).

**Table 6:** Pairwise correlations coefficients between marker additive effects for combination of diseases

Population	Ki3/H100 ^1^(N = 213)		Ki3/Oh7B N = 212)		NC262/H100 (N = 243)		NC262/Oh7B (N = 192)	
**Disease**	NLB	GLS	NLB	GLS	NLB	GLS	NLB	GLS
**SLB**	0.12*	0.37***	0.00	0.06	0.14*	0.17*	−0.09	0.14*
**NLB**	.	0.19**	.	0.22**	.	0.18**	.	−0.02

Population	NC304/H100 (N = 239)		NC304/Oh7B (N = 209)		NC344/H100 (N = 246)		NC344/Oh7B (N = 210)	
**Disease**	NLB	GLS	NLB	GLS	NLB	GLS	NLB	GLS
**SLB**	0.05	0.23***	0.19**	0.15*	0.08	0.26***	0.06	0
**NLB**	.	0.06	.	0.17	.	0.14*	.	0.28***

1 Number of markers segregating in each population (N). Correlations between marker effects were estimated using all the markers selected by the stepwise regression procedure and used in the QTL analysis. Significant p-values are represented by ***, **, *, and * at a level of 0.001, 0.01, 0.05, and 0.1 respectively.

**Figure 3:**
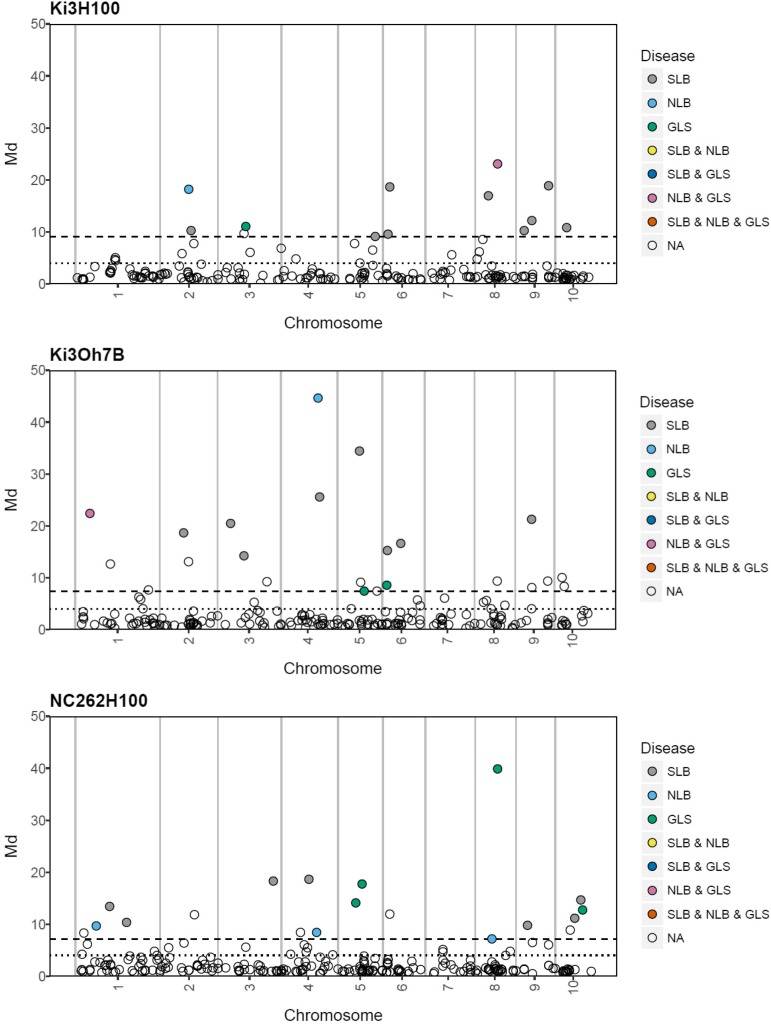

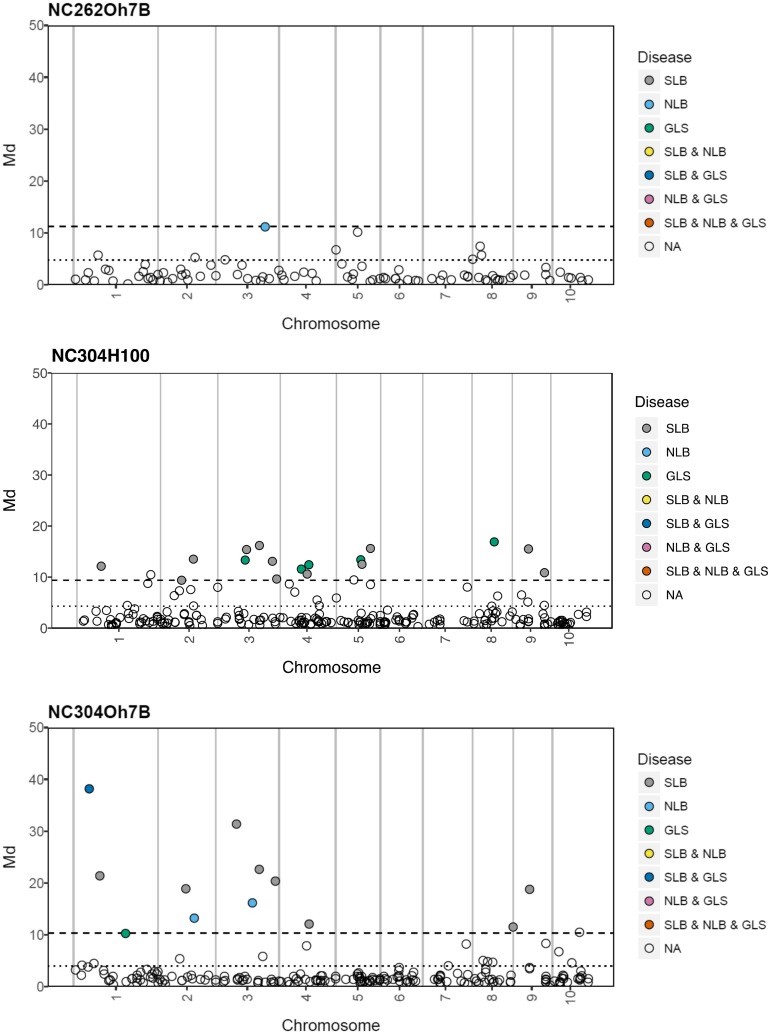

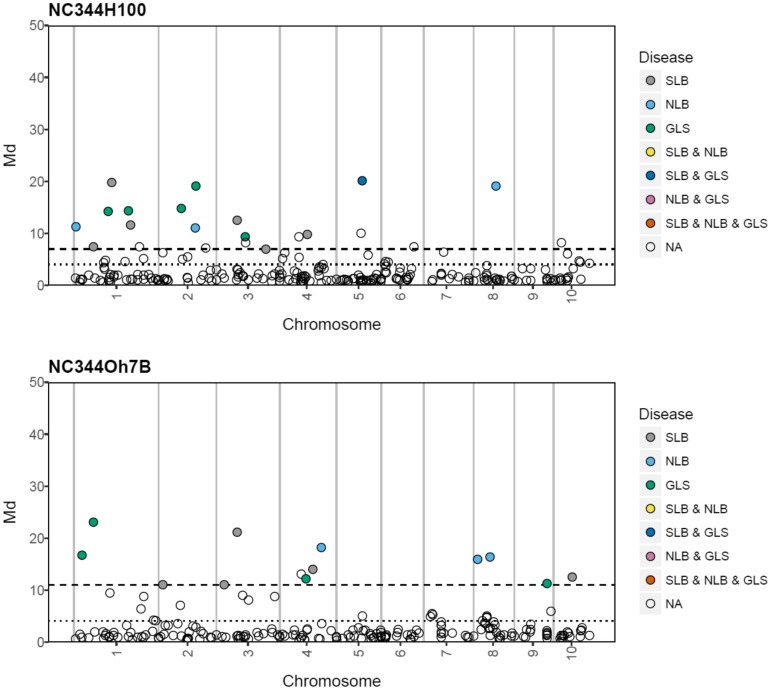
Multivariate outlier markers detected by the *Md* composite statistic. The dotted line corresponds to a 1% FDR for the *Md* value. Points are color coded according to whether the marker LOD score exceeded the disease-specific permutation threshold from QTL analysis for one or more diseases. The dashed line marks the *Md* value at which the minimum LOD threshold for a single disease exceeds the lowest threshold (thresholds were specific to each disease).

In order to identify additional markers potentially associated with MDR, a composite statistic, *Md* (Rousseeuw 1985, Rousseeuw and Zomeren, 1990; Lotterhous *et al.* 2017), which accounts for covariance between the QTL test results for each trait was used (the *Md* statistic was used because the IciMapping software does not include a multivariate QTL test statistic). Similar to a standard multivariate test, significant multi-trait marker associations can occur when a marker for any one trait or multiple traits deviate. Indeed, all of the significant marker associations identified by single disease QTL analysis (Figure 2) were also declared significant according to the *Md* statistic (1% FDR). However, several additional markers with relatively low LOD scores for individual traits had significantly large *Md* values (Figure 3). In total, 246 of the 1652 computed marker *Md* values were significant (File S10). Across all populations, the additive effects of these markers were correlated as expected for MDR between SLB and GLS (0.18) and NLB and GLS (0.15), but the correlation was essentially zero (-0.01) between SLB and NLB (instead, specific populations were positively correlated and others were negatively correlated). Therefore, *Md* helped highlight potential MDR loci, but inspection of the individual test statistic results and more highly replicated validation studies are needed to confirm the association of specific loci with MDR. Moreover, due to intricacies in QTL detection that may result in neighboring markers being declared significant despite the same variant being causal, or because MDR is due to linkage and not pleiotropic effects, loci with closely linked marker associations also represent good candidates for further investigation. Therefore, the co-localization of QTL for different diseases within and across populations was also examined.

QTL for each disease were identified at the same loci in different populations (Table S3). For instance, fourteen QTL for SLB co-localized in at least two populations, and two of them were detected in four of ten populations (on chromosome 3, at 66.37-68.94 cM, and on chromosome 9 at 59.01-60.93). For NLB, six QTL co-localized in two populations; and for GLS, six QTL co-localized in at least two populations (Table S4).

When QTL were localized to maize bins (Davis *et al.* 1999, Table S4), several genomic regions were associated with resistance to more than one disease in the same population. QTL for SLB and GLS co-localized in bins 1.05, 1.06, 3.04, 4.05, 6.01, and 10.04. For SLB and NLB, three QTL co-localized in bins 1.05, 2.05, and 4.05. For NLB and GLS, three QTL co-localized in bins 1.03, 2.05, and 8.03. One QTL in bin 5.04 (chromosome 5 at 90.96-95.23 cM) was associated with resistance to all three diseases in population NC344/H100; in this case, the allele derived from the MDR resistant donor increased susceptibility rather than resistance (Table S4). These results reinforce prior findings (*e.g.*, Tanksley *et al.*, 1996, Balint-Kurti *et al.*, 2008b, Balint-Kurti, *et al.*, 2007, Kump *et al.* 2011, Benson *et al.* 2015) that favorable alleles can be found in lines with unfavorable phenotypes. In addition, we noted numerous QTL associated with one disease in one population and with a second or third disease in a different population (Table S4).

Previous studies reported MDR QTL in bins 1.06, 9.02-9.03 for all three diseases, in bins 1.08-1.09, 2.04, 3.04, and 10.05 for SLB and GLS, in 1.04 and 2.02 for NLB and GLS and in 6.01and 8.05 for NLB and SLB (Balint-Kurti *et al.*, 2010; Belcher *et al.*, 2012; Chung *et al.*, 2011; Zwonitzer *et al.*, 2010). Also, a GST gene in bin 7.02 was associated with variation in MDR (Wisser *et al.*, 2011). In this study, some QTL co-localized with those reported previously, but the association with all three diseases was not observed. The one at bin 1.06, reported as MDR for three diseases, was identified for SLB and GLS in this study. The QTL at 9.03, also reported previously to confer resistance to three diseases, was identified in this study for SLB and NLB. QTL previously identified in bins 1.08-1.09 and 3.04 for SLB and GLS, were also identified in this study to confer resistance to SLB and GLS. Most of MDR QTL (for three or two diseases) detected in this study are novel.

In summary, using eight populations comprising more than 1611 lines, we identified a large number of single disease resistance QTL, and several genomic regions with effects on multiple diseases (Figures 2 and 3). Using several approaches, we demonstrated that alleles associated with resistances to these three diseases tend to co-localize in the genome and confer MDR (allelic effects consistently increase resistance). It is likely that in some cases these are due to the co-localization of alleles with disease specific effects and in others to the pleiotropic effects of single alleles. These populations will likely be of utility in mapping other traits including disease resistance. One recent study used some of these populations to map loci for resistance to Goss’s Wilt (Cooper *et al.* 2018). It will also be helpful to develop higher density genotypic datasets for these populations. We are currently working on fine mapping some of these loci to distinguish between these possibilities.
